# Circulating Branched Chain Amino Acid Concentrations Are Higher in Dairy-Avoiding Females Following an Equal Volume of Sheep Milk Relative to Cow Milk: A Randomized Controlled Trial

**DOI:** 10.3389/fnut.2020.553674

**Published:** 2020-11-05

**Authors:** Amber M. Milan, Linda M. Samuelsson, Aahana Shrestha, Pankaja Sharma, Li Day, David Cameron-Smith

**Affiliations:** ^1^The Liggins Institute, The University of Auckland, Auckland, New Zealand; ^2^AgResearch Ltd, Grasslands Research Center, Palmerston North, New Zealand; ^3^Riddet Institute, Palmerston North, New Zealand

**Keywords:** ovine milk, bovine milk, protein digestion, milk alternative, essential amino acids, postprandial, adult nutrition

## Abstract

**Background:** Intolerances to bovine dairy are a motivating factor in consumers seeking alternate—or replacement—dairy beverages and foods. Sheep milk (SM) is an alternate dairy source, with greater protein, although similar amino acid composition compared to cow milk (CM). Studies are yet to address the appearance of circulating amino acids following consumption of SM, relative to CM, in humans.

**Objective:** To clinically determine the appearance of branched chain amino acids, and other amino acids, in circulation in response to equal servings of SM and CM, in females who avoid dairy products.

**Design:** In a double-blinded, randomized, cross-over trial, 30 self-described dairy avoiding females (20–40 years) drank 650 mL of SM or CM that were reconstituted from the spray dried powders (30 and 25 g in 180 mL water, respectively) on separate occasions, following an overnight fast. After reconstitution, the energy and protein provided by SM was higher than for CM (2,140 vs. 1,649 kJ; 29.9 vs. 19.4 g protein); content of branched chain amino acids (BCAAs) were 10.5 and 6.5 mg**·**mL^−1^, respectively. Blood samples were collected at fasting and at regular intervals over 5 h after milk consumption. Plasma amino acids were measured by HPLC.

**Results:** 80% of subjects self-identified as lactose intolerant, and the majority (47%) “avoided drinking milk” “most of the time”. SM resulted in greater plasma appearance of BCAAs at 60 min (641.1 ± 16.3 vs. 563.5 ± 14.4 μmol·L^−1^; *p* < 0.001) compared with CM. SM similarly resulted in elevated postprandial concentrations of the amino acids lysine, methionine, and proline, particularly at 240 min (time × milk interactions *p* = 0.011, 0.017, and *p* = 0.002, respectively). Postprandial increases in plasma alanine concentrations were sustained to 120 min after CM (time × milk interaction *p* = 0.001) but not after SM, despite greater quantities provided by SM.

**Conclusions:** SM is a rich source of protein, and relative to CM, provides a greater quantity of BCAAs, with a corresponding elevation of the postprandial circulating BCAA response. SM is therefore a possible dairy alternative of benefit to those who need to increase total protein intake or for individuals with heightened protein requirements.

**Unique Identifier and Registry**: https://www.anzctr.org.au/Trial/Registration/TrialReview.aspx?id=375324, identifier U1111-1209-7768

## Introduction

Avoidance of dairy products is prevalent among some populations. In Australia, dairy avoidance may be practiced by 17% of the population, of whom the majority (13%) avoid dairy on the basis of self-reported negative reactions to dairy products (1). This trend is evident globally (2, 3), where perceived or actual intolerance to cow milk (CM)—either due to lactose or other components of milk (4)—influences consumption behaviors (5, 6). Dairy products are recognized as an important source of nutrients (7, 8), including calcium and high quality protein (7), essential for growth and development. Nutritionally, dairy avoidance in the context of a Western diet has the potential to increase the risk of nutrient insufficiencies, contributing to poor health in the form of low bone mineral density and even chronic illness (8, 9). In populations for whom dairy products can be a rich source of energy and protein, such as young children (10) or the elderly (11), it is important to identify suitable dietary alternatives to bovine dairy. While non-dairy alternatives exist, these are often not nutritionally equivalent to dairy, increasing the risk of dietary deficits of key dairy-derived nutrients for dairy avoiders (9). For instance, certain plant-based milks are lacking in essential amino acids (12), with plant proteins contributing less protein accretion than milk proteins (13).

Sheep (ovine) milk (SM), while compositionally different to CM, is an alternate ruminant milk which maintains many of the key nutritional features of bovine milk. Anecdotal evidence that milk from other ruminants (goat and sheep) is easier to digest than bovine milk (14), particularly for those with intolerance to dairy products (15), has been cited to support the use of SM as a more suitable alternative to CM. Compared to cow milk, sheep milk has a higher total solids content, accounted for by greater protein (6.2 vs. 3.2 %) and fat (7.9 vs. 3.6 %) content (16). On a weight (mg/g protein) basis, sheep milk has similar essential amino acid (AA) content as bovine milk (17). However, there are sequence differences of key proteins, including α_s1_-, α_s2_-, β-, and κ-caseins (18) and whey proteins (19). This leads to the structural and physiochemical properties of SM when milk is acidified (e.g., in processing or in human stomach) (16). These types of physicochemical effects have been shown to influence digestion kinetics for bovine milks (20), a factor which impacts on AA appearance in circulation (21, 22) and postprandial protein accretion (22, 23). Although in-depth descriptions of SM digestion kinetics are limited (24, 25), a rodent model demonstrated higher ileal amino acid digestibility in rats fed SM relative to CM, resulting in greater circulating concentrations of leucine, lysine, and methionine (26). Thus, SM with greater protein composition and greater ileal digestibility than CM may offer relative advantages in meeting protein needs. This may be of particular importance for those with low protein intake or who have greater protein requirements, including those who habitually avoid bovine dairy. Studies are yet to address the differences in protein digestion and AA appearance in human subjects.

The aim of this study was to compare, on a “portion-for-portion” basis, the appearance in circulating BCAA (i.e., leucine, valine and isoleucine) following ingestion of sheep or cow milk, and further to compare the appearance of other AA in circulation. CM composition is consistent throughout the year due to continual production and large volumes. However, SM is currently a niche product, with a seasonal production pattern in New Zealand. The composition of SM varies throughout the season, notably with an increase in fat and protein content toward the end of the season (16, 27). In addition, there is a difference in milk composition between producers (27), across sheep breeds (28, 29) and in geographical regions (17, 28, 29). Thus, spray dried powdered milk was selected as a shelf stable milk preservation technique that was suitable for both sheep and cow milk. To improve the generalisability of the results, reconstituted sheep milk powder from two different producers and commercially available reconstituted cow milk powder were used as the test drinks in this study.

We hypothesized that circulating BCAAs and other AA concentrations would be greater following the ingestion of SM compared to CM.

## Methods

### Experimental Design

The study was a double-blinded, randomized and crossover study, with equal allocation ratio and an equivalence framework. The study was conducted at the Clinical Research Unit, Liggins Institute, University of Auckland between July and November 2018. Postprandial appearance of BCAA at 60 min (peak appearance) was compared between sheep and cow milk as the primary outcome. Secondary outcomes of the postprandial appearance over 4 h of all amino acids were also compared. Additional secondary outcomes of lipid responses, lactose malabsorption, subjective appetite and digestive comfort were also collected, but have not been reported here.

The study was conducted according to the guidelines laid down in the Declaration of Helsinki and all procedures involving human subjects were approved by the New Zealand Health and Disability Ethics Committees (Reference no. 18/NTB/92). The trial was prospectively registered with the Australian New Zealand Clinical Trials Registry (ACTRN12618001030268). Written informed consent obtained from eligible participants prior to study commencement.

### Participants

**Figure 1:**
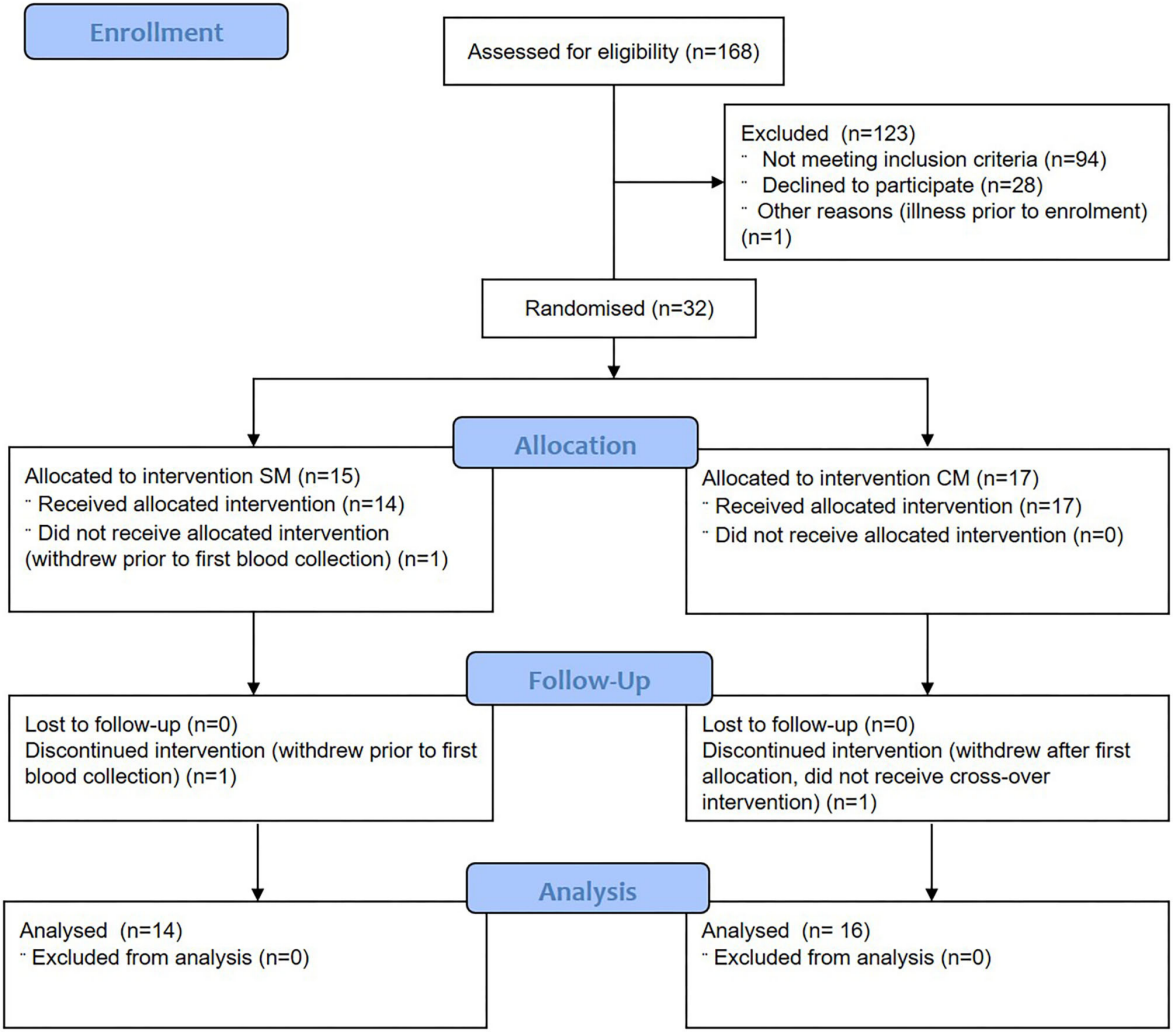
Consolidated Standards of Reporting Trials flow diagram of participant eligibility, enrolment, randomization, follow-up, and analysis. SM, sheep milk; CM, cow milk.

Females (*n* = 32) aged 20–40 years were recruited using print and electronic advertisements. Two subjects withdrew prior to the completion of the protocol (Figure 1). Participants were required to self-describe to “avoid drinking milk” as a binary classification (yes/no) and have a BMI between 18 and 28 kg/m^2^. Participants were not eligible if they had a known allergy to milk, or known gastrointestinal (celiac, or inflammatory bowel disease) or metabolic disease, or were currently taking medications expected to interfere with normal digestive and metabolic processes including proton pump inhibitors, laxatives, antibiotics or prebiotics (within last 3 months). Participants with pre-existing cardiovascular diseases or self-reported alcohol intake >28 units per week were also not included.

### Study Procedures

Following determination of eligibility, subjects were randomized to receive either sheep milk or cow milk in a cross-over manner at least one week apart. Randomization sequences were computer-generated by www.randomizer.org (30). Allocation was implemented through sealed envelopes. Investigators and participants were blinded to treatment identity and until primary outcome data analysis was complete.

Subjects were asked to attend the Clinical Research Unit on two occasions, separated by at least one week. Prior to the visits, subjects completed a validated lactose intolerance screening questionnaire (31), along with questions about habitual dairy consumption patterns. On the day preceding each visit, subjects were asked to avoid foods high in fat, alcohol, caffeine and taking part in any strenuous exercise. Subjects were provided with a standardized low-fiber, low-fat meal on the day preceding the visit to consume as their evening meal.

Subjects arrived the following morning, after an overnight fast, excluding water, from 10 pm the night prior. A venous cannula was inserted, and a fasting blood sample collected. Subjects then consumed 650 mL of the assigned milk, within 10 min.

Following milk ingestion, blood samples were collected at 15 min intervals for the first 90 min, and then hourly starting at 2 h for 4 h post-ingestion. During the 4 h, subjects refrained from the consumption of any additional food or drink except water.

Blood pressure was assessed at fasting at each acute visit by automatic blood pressure monitor (Heart Sure BP100, Omron, Kyoto, Japan). Height (by stadiometer to the nearest 0.1 cm; Holtain Ltd., Crymych, Dyfed, UK), weight (by digital weighting scale to the nearest 0.1 kg; Tanita^®^ 1582 Medical Scale, Wedderburn, Auckland, New Zealand) and waist circumference (by non-flexible tape measure) were measured on the first visit only by standard procedures in the fasted state after voiding.

### Blood Collection

Venous blood samples were collected in EDTA containing vacutainers (Becton Dickinson & Company, Mount Wellington, New Zealand), and plasma was removed after centrifugation at 2,000 x *g* for 15 min at 4°C and frozen at −80°C prior to analyses.

### Questionnaires

Demographic information about dairy consumption behaviors (avoidance, restriction, frequency of symptoms experienced) was collected using categorical scales. The frequency of milk consumption was assessed as serves per day, and habitual frequency by food frequency questionnaire (FFQ) category scale from the EPIC-Norfolk FFQ (32). Perceived lactose intolerance was assessed using a validated visual analog scale by the sum of abdominal rumbling, cramps, flatulence, diarrhea, and vomiting (31).

### Study Treatments

Whole sheep milk powder was sourced from Blue River Dairy (batch no. F2125/HC08) and Spring Sheep Milk Company (batch no. MAN: NOV17-JAN18) and these were blended 1:1 prior to weighing and reconstitution for both consumption and all subsequent milk analysis. Whole cow milk powder was commercially sourced from NZMP (New Zealand Milk Products, Fonterra Co-Operative Group, Auckland, New Zealand). Milk powders were stored frozen (−20°C) prior to use.

Test drinks made from reconstituted whole cow (CM) and whole sheep milk (SM) powder were provided in 650 mL quantities. Milks were prepared from milk powder the evening prior to the visit using heated (30°C) filtered water, shaken vigorously, then stored overnight at 4°C. Pre-weighed portions of cow milk powder or sheep milk powder (81 or 98 g, respectively) were reconstituted in 585 mL water. The reconstitution was according to the manufacturer's instructions, providing a similar sensory profile between milks, and to approximate the proportional solids content in an equal volume of fresh liquid milk. Milks were prepared in opaque plastic drinkware and served chilled. No sensory masking of products was used.

The nutrient and total amino acid composition of each milk (cow and blended sheep milk, respectively) are provided in Tables 1, 2, respectively. Reconstituted SM was higher in total energy, fat, protein, total solids, and solids non-fat, but slightly lower in lactose than CM. With a higher total protein content than CM, the total amino acid content of SM was also greater. Proportions of amino acids as % content were generally similar (within 5%), aside from tryptophan and alanine which are present in 18 and 10% higher proportion in SM compared to CM, respectively.

**Table 1:** Proximate composition of 650 mL of the sheep and cow milk test drinks.

**Component**	**Cow milk**		**Sheep milk**	
	**Amount**	**%**	**Amount**	**%**
Total energy (kJ)	1649.3	-	2140.4	-
Fat (g)	21.3	3.3	33.4	5.1
Protein (g)	19.4	3.0	29.9	4.6
Lactose (g)	33.3	5.1	24.9	3.8
Total solids (g)	79.0	12.2	91.7	14.1
Solids non-fat (g)	57.7	8.9	60.3	9.3

*Test drinks were prepared from 25 g whole cow milk powder or 30 g whole sheep milk powder reconstituted in 180 mL water, respectively, per a total reconstituted volume of 200 mL*.

**Table 2:** Amino acid content of sheep and cow milk.

	**Cow milk**		**Sheep milk**	
**Amino Acid**	**mg·mL^**−1**^**	**% of total amino acids**	**mg·mL^**−1**^**	**% of total amino acids**
**ESSENTIAL AMINO ACIDS**				
Leucine	2.95	9.35	4.85	9.59
Lysine	2.50	7.92	4.12	8.15
Valine	1.94	6.14	3.17	6.26
Isoleucine	1.59	5.03	2.48	4.90
Phenylalanine	1.45	4.61	2.30	4.55
Threonine	1.40	4.43	2.25	4.44
Histidine	0.84	2.66	1.35	2.66
Methionine	0.67	2.12	1.13	2.22
Tryptophan	0.41	1.29	0.78	1.53
**NON-ESSENTIAL AMINO ACIDS**				
Glutamic acid^†^	6.32	20.02	9.64	19.04
Proline	3.04	9.63	4.90	9.68
Aspartic acid^†^	2.39	7.57	4.06	8.03
Serine	1.73	5.49	2.54	5.02
Tyrosine	1.41	4.45	2.24	4.42
Alanine	1.06	3.34	1.86	3.68
Arginine	1.06	3.37	1.61	3.18
Glycine	0.59	1.86	0.96	1.90
Cystine	0.23	0.72	0.37	0.73

† *Results for aspartic acid and glutamic acid may include contributions of asparagine and glutamine, respectively, converted during hydrolysis*.

### Analysis Methodology

#### Chemicals

Sodium acetate trihydrate (pro analysis), acetic acid (glacial), acetonitrile (HPLC grade), and methanol (HPLC grade) were purchased from Merck KGaA (Darmstadt, Germany). Disodium hydrogen orthophosphate heptahydrate (98.0–102.0%), ethylenediamine tetraacetic acid (EDTA) (>99%), and orthophosphoric acid (>85%) were purchased from VWR Chemicals BDH (Radnor, PA, USA). Triethylamine (>99%), DL-norleucine (98%), L-asparagine (>98%), L-glutamine (>99%), L-tryptophan (>98%), DL-dithiothreitol (>985), and phenylisothiocyanate (>99%) were purchased from Sigma-Aldrich (St. Louis, MO, USA).

The following reference materials were purchased from Sigma-Aldrich: Amino acid standard solution AAS18 (2.5 μmol mL^−1^ of 18 proteinogenic amino acids in 0.1 N HCl), Amino acid standard solution A6407 (2.5 μmol mL^−1^ of 26 physiological amino acids in 0.1 N HCl), Amino acid standard solution A6282 (2.5 μmol mL^−1^ of 14 physiological, basic amino acids in 0.1 N HCl).

A proteinogenic amino acid standard consisting of amino acid standard solution AAS18 and 0.05 μmol mL^−1^ of L-asparagine, L-glutamine and L-tryptophan was prepared. A composite physiological amino acid standard was prepared from equal volumes of amino acid standard solutions A6407 and A6282.

#### Biochemical Analysis

Plasma free amino acids were analyzed by the AgResearch Analytical Laboratory (Palmerston North, New Zealand) using the Pico-Tag method (33). Each plasma sample (500 μL) was mixed with 25 μL of 80 mmol L^−1^ dithiothreitol in 200 mmol L^−1^ phosphate buffer as an antioxidant, and 10 μL of 10 mmol L^−1^ norleucine as an internal standard. The mixture was filtered through a 2-mL Vivaspin 500 centrifugal concentrator (10 kDa MWCO, Bio-Strategy, Auckland, NZ) and centrifuged (90 min, 10,000 x *g*, 4°C). The filtrate (50 μL) was lyophilized for 1 h in a freeze drier (Flexi-Dry, FTS Systems, Stone Ridge, NY, USA) and then reconstituted in 20 μL freshly prepared methanol/1 M sodium acetate/triethylamine (2:2:1) and lyophilized again (16 h). The dried sample was reconstituted in 20 μL derivatization solution (freshly prepared methanol/Milli-Q water/triethylamine/phenylisothiocyanate, 7:1:1:1), and incubated at room temperature for 20 min and then lyophilized (2 h). The dried sample was finally reconstituted in 200 μL of the sample diluent (0.71 mg mL^−1^ disodium hydrogen orthophosphate in Milli-Q water, pH adjusted to 7.40 with 10% phosphoric acid, diluted to 5% v/v in acetonitrile). The sample was centrifuged (5 min, 12,000 x *g*, room temperature), and the supernatant was used for the HPLC analysis.

Amino acids were resolved on a PicoTag^®^ For Free Amino Acid Analysis column (60 Å, 4 μm, 300 mm x 3.9 mm) (Waters Corporation, Massachusetts, USA) using a LC-10ADvp instrument (Shimadzu Corporation, Kyoto, Japan). The extract 50 μL was injected onto the column, which was held at 46°C, and eluted over a 90-min gradient with a flow rate of 1 mL**·**min^−1^. UV detection was carried out at 254 nm. The mobile phase was a mixture of buffers A and B. Buffer A consisted of 1,964 g stock buffer (38.16 g sodium acetate trihydrate dissolved in Milli-Q water and made up to 4 L, pH adjusted to 6.50 with 10% v/v acetic acid), 35 g acetonitrile, and 500 μL 10 mM EDTA. Buffer B consisted of 900 mL acetonitrile, 800 mL Milli-Q water, and 300 mL methanol. The gradient elution programme was as follows: held at 100% A (0–13.5 min); 0–2% B (13.5–24 min); 2–6% B (24–30 min); 6–28% B (30–50 min); 28–31.5% B (50–62 min); held at 31.5% B (62–70 min); 31.5–100% B (70–70.5 min); held at 100% B (70.5–74.5 min); 100–0% B (74.5–75 min); held at 100% A (75–90 min).

Analytical batches consisted of a blank, a derivatization blank, the proteinogenic amino acid standard, and the composite physiological amino acid standard, followed by up to eight samples injected sequentially. Standards were run after every eight sample injections. The derivatization blank was used to verify that the observed response of each analyte was not affected by the derivatization matrix. Quantification of amino acids was based on the mean standard response of each analyte. Typical chromatograms of human plasma are shown in Supplemental Figure 1.

Plasma glucose was measured using a Roche Cobas c311 by enzymatic colorimetric assay (Roche Diagnostics, Mannheim, Germany). Plasma insulin was measured using a Cobas e411 immunoassay analyser (Roche Diagnostics, Mannheim Germany).

#### Milk Compositional Analysis

Cow or sheep milk powders (12.5 and 15.0 g, respectively) were reconstituted in 90 mL MilliQ water for 20 min at room temperature. The sheep milk powder was blended 1:1 from the two suppliers prior to weighing and reconstitution as described above. The dissolved powders were then kept at 4°C overnight to reach full hydration. The samples were warmed to 40°C in a water bath in the following day. The proximate composition analysis was performed using a MilkoScan FT1 (FOSS, Denmark) analyzer using the default milk mosaic software. Amino acids content of the milk powders was determined by the AgResearch Analytical Laboratory (Palmerston North, New Zealand). Milk powders were hydrolysed using 7.5 M hydrochloric acid, performic acid oxidation, and alkaline hydrolysis with 4.67 M sodium hydroxide for acid stable amino acids, sulfur amino acids and tryptophan, respectively, prior to analysis. All amino acids were analyzed using a sodium-based ion exchange chromatography with post-column derivatisation by ninhydrin with absorbance readings at 570 and 440 nm (modified AOAC methods 994.12, 994.12, and 988.15, respectively).

### Statistical Analysis

A sample size of 30 subjects was determined to be required to detect a 20% difference in peak amino acid concentrations with a power of 90% using α 0.05. This was based on previously reported 60 min concentrations of 608 μmol·L^−1^ with a standard deviation of 198 μmol/·L^−1^ (34).

Statistical analyses were performed with SPSS version 25 (SPSS, IBM Corporation, Armonk, NY, USA). Continuous data are presented as mean ± SEM. Study outcomes were analyzed on a per protocol basis. Incremental area under the curve (iAUC) was calculated using the trapezoidal method, correcting for baseline concentrations. Outliers in amino acid data were identified as greater or <Q3+3IQR. Multiple imputation was used for values missing completely at random, as the mean of 5 iterations. Values lower than the limit of quantification were imputed at 50% of the limit of detection.

Continuous variables were analyzed using parametric tests. Single factor comparisons, such as iAUC, were made using Student's *t*-test with the null hypothesis that there is no difference between the test drink treatments. The null hypothesis was rejected, and the difference between treatments statistically significant, if P < α and t > t_crit_. All outcomes with multiple factors were analyzed by repeated factor generalized linear model with milk and time compared within-subject and adjusted for multiple comparisons using a Sidak Holm adjustment. The Huynh-Feldt correction was used where Mauchly's sphericity test failed. Alpha was set at *P* < 0.05 for all tests.

Heat maps were created using R software version 2.15.2 (35) with gplots (heatmap.2), RColorBrewer and colorRamps packages (R Development Core Team).

## Results

### Demographics

Thirty females completed the study. All subjects had anthropometric and biochemical values within a healthy range (Table 3).

**Table 3:** Baseline participant characteristics.

**Measure**	**Mean ± SEM**
Age (years)	24.4 ± 1.1
BMI (kg/m^2^)	23.3 ± 1.1
Waist circumference (cm)	77.2 ± 1.1
Glucose (mmol/L)	4.9 ± 0.1
Blood pressure (mmHg)	
Systolic	107 ± 3
Diastolic	72 ± 1

*Values presented as means ± SEM from fasting samples collected on the first visit only, aside from glucose and blood pressure, which represent the mean of both visits*.

Although all subjects indicated they “avoided drinking milk” (as a binary classification to determine eligibility), the majority of subjects reported avoiding dairy “most of the time” (*n* = 14; 47%) or “sometimes” (*n* = 12, 40%), with only four subjects (13%) indicating “seldom,” and none (0%) responding “always.” Similarly, the majority classified their restriction of dairy as “eat less than desired” (*n* = 26, 87%), with a minority indicating they “avoid altogether” or have “no restriction” (*n*= 2, 7% each).

Most subjects classified their self-reported symptoms from dairy as causing “occasional” (*n* = 19, 63%), or “usual” (*n* = 9, 30%) symptoms, with two subjects (7%) reporting “no problem.” When screened for lactose intolerance, 24 (80%) subjects were identified as lactose intolerant (score >70), with a mean perceived symptom score of 168 ± 17 mm.

Fourteen subjects reported consuming a maximum of 250 mL milk per day in any form (i.e., including with tea, coffee, cereals, etc.; 47%), while the majority reported no daily consumption (*n* = 16, 53%).

### Postprandial Amino Acid Response

**Figure 2:**
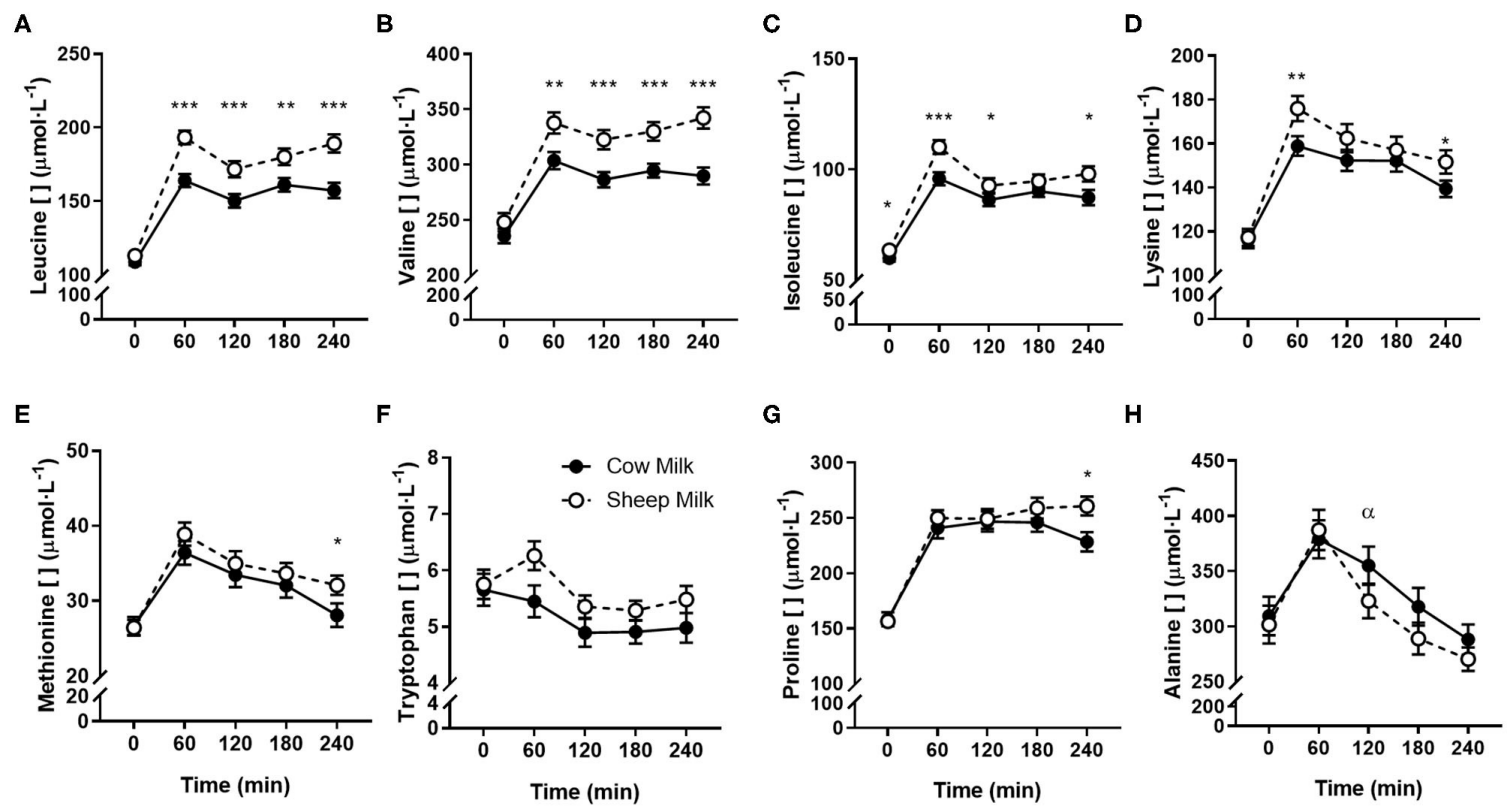
Postprandial changes in plasma amino acids differing between sheep and cow milk following ingestion. Values presented as means ± SEM for leucine **(A)**, valine **(B)**, isoleucine **(C)**, lysine **(D)**, methionine **(E)**, tryptophan **(F)**, proline **(G)**, and alanine **(H)**. There was a main effect of milk for tryptophan (*p* = 0.012) and a time × milk interaction for all other presented amino acids (*p* < 0.05 each, respectively). ^*^p < 0.05, ^**^p < 0.01, ^***^p < 0.001 denote statistical significance between sheep (◦) and cow (•) milk; α denotes significant changes from baseline after cow milk (Sidak corrected *post hocs*).

Peak plasma BCAA concentrations (at 60 min) were greater following SM than CM ingestion (641.1 ± 16.3 vs. 563.5 ± 14.4 μmol/L; *p* < 0.001). Individually, all plasma BCAA responses differed between milks (Figure 2). Leucine and valine concentrations were higher following SM at all postprandial time points (*p* = 0.001 and *p* < 0.001 time × milk interaction, respectively; *p* < 0.01 for all postprandial time point pairwise comparisons; Figures 2A,B). Isoleucine concentrations were similarly higher following SM (*p* = 0.031 time × milk interaction), apparent postprandially but also at baseline (*p* < 0.05 all pairwise comparisons at 0, 60, 120, and 240 min; Figure 2C). Similarly, the iAUC for BCAAs in circulation was higher for SM than CM (*p* < 0.05; Table 4).

**Table 4:** Amino acid iAUC following sheep and cow milk.

**Amino acid^a^**	**Cow milk**	**Sheep milk**	***p* value^b^**
Isoleucine	6,367 ± 336	7,465 ± 501	0.012
Leucine	10,417 ± 572	14,627 ± 901	<0.001
Valine	12,307 ± 702	17,606 ± 1,225	<0.001
Lysine	7,604 ± 605	9,652 ± 705	0.008
Methionine	1,361 ± 144	1,860 ± 170	<0.001
Threonine	3,520 ± 441	4,602 ± 650	0.073
Phenylalanine	1,713 ± 157	1,946 ± 187	0.140
Histidine	1,658 ± 257	1,958 ± 279	0.423
Tryptophan	−124 ± 43	−29 ± 45	0.076
Proline	17,639 ± 891	20,476 ± 1,087	0.003
Glutamic acid	1,309 ± 462	2,025 ± 319	0.055
Aspartic acid	55 ± 53	198 ± 41	0.024
Tyrosine	3,991 ± 213	4,097 ± 322	0.733
Asparagine	2,065 ± 185	2,340 ± 253	0.220
Glutamine	14,304 ± 1,700	13,122 ± 1,585	0.536
Serine	2,754 ± 321	2,519 ± 371	0.499
Arginine	1,541 ± 238	1,824 ± 220	0.195
Alanine	6,760 ± 2,046	4,716 ± 1,828	0.305
Cystine	−29 ± 12	−13 ± 10	0.243
Glycine	−3,241 ± 654	−4,768 ± 621	0.037^c^
Ornithine	1,922 ± 178	2,209 ± 206	0.169
Citrulline	−1,511 ± 112	−1,280 ± 120	0.124
Hydroxyproline	−91 ± 41	−126 ± 58	0.615
1-Methylhistidine	−132 ± 52	−270 ± 94	0.140
3-Methylhistidine	−82 ± 25	−38 ± 27	0.192
αAminobutyric acid	61 ± 244	85 ± 433	0.959
Taurine	−308 ± 328	51 ± 388	0.441

a *Values presented as means ± SEM in μmol·L^−1^**·**min^−1^*.

b *Significance analyzed by Student's t-test*.

c *Although p < 0.05 for Gly, t < t_crit_ and the null hypothesis was not rejected*.

**Figure 3:**
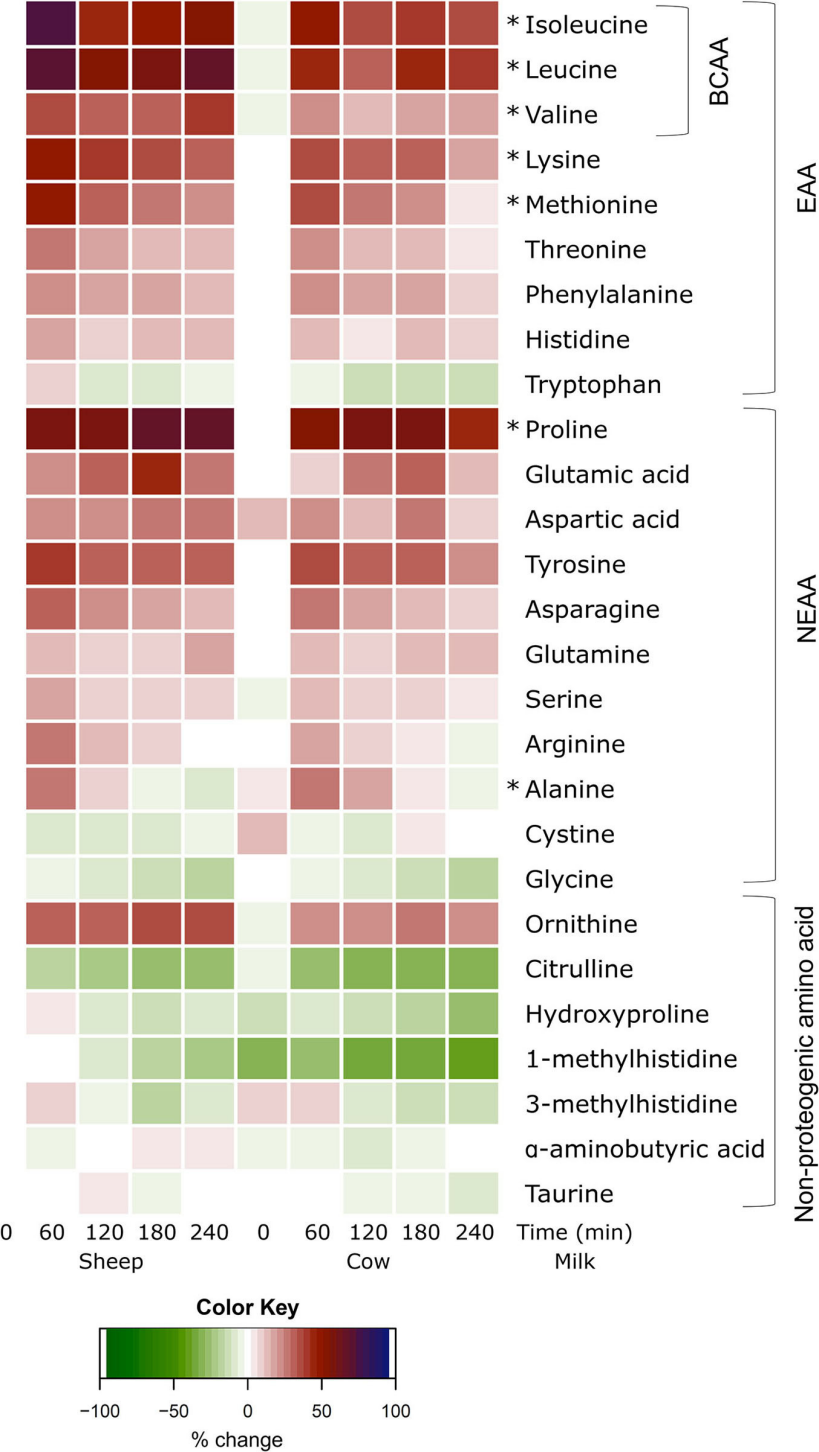
Heat map of postprandial changes in individual plasma amino acids after sheep and cow milk ingestion. Values are presented as mean percent changes relative to concentrations at fasting prior to sheep milk ingestion. White represents a 0% change from sheep milk fasting concentrations. Red represents a 50% increase; blue represents a 100% increase; green represents a decrease. “0”: timepoint 0 min; “60”: timepoint 60 min; “120”: timepoint 120 min; “180”: timepoint 180 min; “240”: timepoint 240 min. Amino acids are grouped descending from the top by branched-chain amino acids (BCAA), essential amino acids (EAA), non-essential amino acids (NEAA), and non-proteogenic amino acids. ^*^denotes significant (*p* < 0.05) interaction of time × milk.

All individual amino acid responses are shown as a heatmap (Figure 3) displaying the percentage change from SM fasting concentrations (0 min). All amino acid concentrations changed over time independent of the type of milk consumed (Supplemental Figures 2–4; *p* < 0.05 each, respectively), except for taurine which did not change after milk ingestion (*p* > 0.05 main time effect). The majority increased with milk ingestion, while decreases were observed for tryptophan, cystine, glycine, and all non-proteogenic AA except ornithine and α-aminobutyric acid.

Aside from BCAAs, only the EAAs lysine and methionine, and NEAAs proline and alanine responded differently between milks (*p* = 0.011, 0.017, *p* = 0.002, and *p* = 0.001 time × milk interaction, respectively). Both lysine and methionine concentrations were higher at 240 min following SM than CM (*p* < 0.05 between milks; Figures 2D,E), with lysine concentrations additionally greater at the 60 min peak concentration (*p* = 0.001). Both lysine and methionine iAUCs were similarly greater with SM than CM (*p* < 0.01; Table 4). Tryptophan concentrations were higher with SM but were independent of postprandial time (*p* = 0.012 main milk effect; Figure 2F). Proline concentrations were higher following SM than CM at 240 min (*p* = 0.016; Figure 2G), which was mirrored in the iAUC (*p* = 0.003). Alanine concentrations did not differ between milks at any time point (Figure 2H), as reflected by similar iAUCs (*p* = 0.305), but the rise in alanine concentrations from baseline to 120 min was sustained following CM ingestion (*p* = 0.004) but not SM (*p* > 0.05). Although the iAUC for aspartic acid concentrations was greater for SM than CM (*p* = 0.024), this was not reflected as an interaction of milk and time (*p* = 0.186).

### Postprandial Glycaemic Response

**Figure 4:**
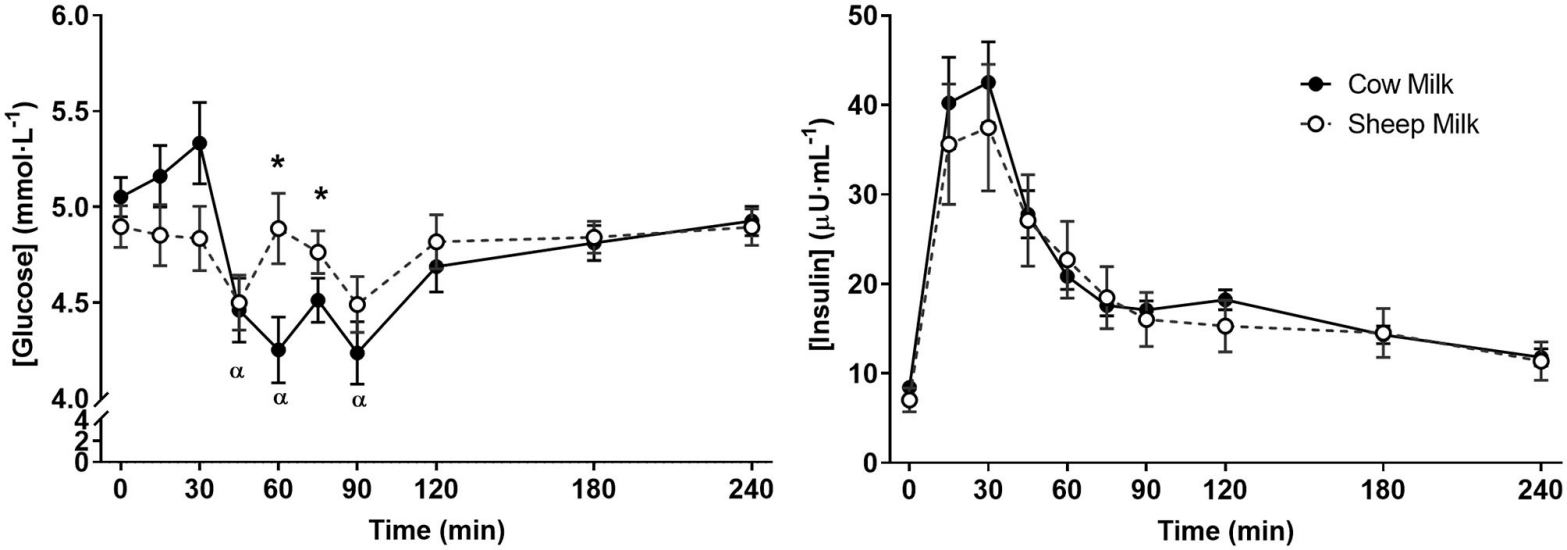
Postprandial concentrations of glucose and insulin following sheep and cow milk. Values represent means ± SEM in mmol·L^−1^ for glucose and μU·mL^−1^ for insulin. There was a time × milk interaction for glucose but not insulin (*p* < 0.05 and *p* > 0.05, respectively). ^*^p < 0.05 denotes statistical significance between sheep (◦) and cow (•) milk; α denotes significant changes from baseline after cow milk (Sidak corrected *post hocs*).

Following milk ingestion, plasma glucose responses to SM and CM were different (time x milk p = 0.002; Figure 4); however, the iAUC did not differ (*p* = 0.145). SM did not impact postprandial glycaemia, but CM resulted in decreased glucose concentrations from fasting to 45, 60, and 90 min (*p* < 0.05 for each) which were lower than those experienced with SM at 60 and 75 min (*p* < 0.05 for each), followed by a return to fasting concentrations at 240 min. In contrast, no differences in insulin response were observed (time x milk *p* > 0.05).

## Discussion

Milk is an important source of dietary protein and minerals, including calcium. However, avoidance of bovine dairy is increasingly frequent, requiring the identification of suitable dietary alternatives to support health. The composition of milk, both with respect to nutrient composition and protein structure, can influence digestive processes and appearance of nutrients in circulation. Despite well-known compositional differences between ruminant milks, including sheep and cow milks, few studies have comparatively described the postprandial impacts on nutrient appearance. The current study addressed this question by comparing the circulating amino acid postprandial responses, following ingestion of equal volumes of SM or CM. The composition of the milks differed, with SM containing a greater quantity of BCAAs and all other AA due to its higher protein content. In response to a single ingestion, there was a greater abundance of BCAAs, methionine, lysine, and proline in circulation. Yet, despite greater compositional abundance of all AAs, no greater circulating abundance of any of the other AAs were observed relative to CM, and in the case of alanine, plasma concentrations were higher after CM.

In the current study, female volunteers consumed a single bolus of 650 mL of either SM or CM. Plasma BCAA concentrations were elevated to a greater degree following SM consumption than CM, notably at peak concentrations (60 min). Peak BCAA concentrations, in particular leucine, have been hypothesized to be important in triggering muscle protein synthesis (MPS) (36–38). From 60 to 240 min, there continued to be higher plasma leucine concentrations following SM ingestion. In addition to the greater abundance of these BCAAs in SM from an equal volume, the higher concentrations in circulation may reflect differences in protein structure. For caseins, sheep milk AA sequences include notable differences to cow milk sequences across α_s1_-, α_s2_-, β_−_, and κ-caseins, which directly influence their enzymatic products and functionality including micelle formation and mineral binding (18). These factors, in addition to macronutrient interactions (including fat globule properties) and macrostructural effects (20), are known to influence digestion rates and AA appearance (20, 39). Irrespective of the underlying reason for higher circulating BCAAs following ingestion with SM, portion-for-portion SM may help to supply sufficient AA acutely and longer term. Indeed, in a rodent model, a similar elevation of leucine, along with lysine and methionine were seen in circulation following long term feeding with SM relative to CM (26). For some populations, such as the elderly, greater acute availability of AAs during peak periods, and also sustained concentrations in circulation following ingestion, may be beneficial for stimulation of MPS (40, 41). Pulse feeding of protein has been shown to improve protein retention in elderly women (40), while the delayed MPS observed following exercise in the elderly (41) may benefit from sustained availability of AAs. Future research should aim to identify whether clinical outcomes relating to elevated AA availability, including measures of MPS, are affected differently with SM ingestion.

Other essential (lysine and methionine) and non-essential (proline) AA concentrations were also elevated more following SM ingestion than CM. While this reflects the greater abundance of these AAs and total protein in SM, the implications of greater postprandial concentrations are less clear than for BCAAs. Indeed, greater intake of specific AAs have been shown to enrich circulating concentrations of these AAs during periods of growth (42). As with BCAAs, adequate postprandial abundance of EAAs during activation of MPS are required for its optimal stimulation (43), but under conditions of adequate abundance, greater postprandial elevations of specific AAs have not been clearly linked to greater MPS stimulation (43). Similarly, for long term health, greater bioavailability of EAAs such as lysine or methionine may be most relevant where these AAs are limited in the diet. Both lysine and methionine are found in short supply in cereal based foods (44), and can be limiting AAs in the diets of populations consuming largely cereal based diets, such as vegetarians (44). When limited in the diet, additional intake of lysine has been shown to have beneficial impacts on anxiety and stress (45), diarrhea (46), or even muscle strength and function in elderly women (47). Hence, in cases of insufficient dietary intake, as in those consuming greater proportions of cereals such as young children (48–51), SM could be an attractive nutrient dense option.

Although most AAs were more abundant in SM than CM, most did not appear in greater quantities in circulation. In the case of alanine, plasma concentrations were actually slightly lower following SM than CM, despite SM providing more alanine. Many ingested AAs are taken up by first pass metabolism by the splanchnic tissues (52), which could explain a lack of difference in circulation for AAs such as glutamine (53) or tryptophan (54), among other AAs (55, 56). Thus, any possible benefit of greater acute intake of these AAs abundant in SM may only be detectable within the gut or liver (27) and may not be apparent in peripheral circulation. Peripheral circulating AAs, in addition to influence from endogenous AA pools (52), are also impacted by gastrointestinal transit (57, 58), or postprandial metabolic responses to meals (58–60). Importantly, the current study did not use the gold standard techniques of isotopically labeled foods to precisely track the fate of all ingested AAs, which limits the current interpretation of the AA appearance differences. Differences in protein peptide structure (18) or physiochemical properties (16, 61) between SM and CM have the potential to influence micelle (62) or curd formation (63), gastric emptying (22), incretin responses (64, 65), or AA accretion independent of total AA content (23). While gastric emptying may impact AA appearance (57, 58), physiochemical properties like curd formation have not been conclusively shown to influence gastrointestinal transit (66–68). Further, comparative descriptions of such properties between SM and CM are limited (63). As such, more detailed descriptions of the physiochemical differences between SM and CM, and their respective influence on digestion dynamics, would be required to attribute circulating AA differences to physical influences of protein structures. Although insulin responses were similar between SM and CM, glucose concentrations decreased slightly with CM but not SM. While this could suggest differences in incretin response (69), gastrointestinal motility or appetite hormone responses were not addressed in this study. Hence, further work is required to understand the specific influence of protein and peptide differences on aspects of digestion and postprandial metabolism impacting circulating AAs.

Comparative assessment of meals with inherently different composition is challenging, as differences may exist not only in the primary nutrient of interest, but across other macro and micronutrients, including total energy. In the current study, equal volumes of milk were selected to provide a comparison of sheep and cow milk on a “portion-for-portion” basis, resulting in greater total protein and specific AA content in SM relative to CM. Although this compositional difference contributes to the greater postprandial rises in AAs observed following SM, it is important to note that fat interactions (70, 71), total energy (72, 73), and volume (74) also impact digestion and possibly nutrient appearance (58, 75) in circulation. Artificially matched formulations to provide equivalent protein and/or AA content would similarly alter or eliminate other compositional differences inherent between milks, which contribute to the overall postprandial response. However, it should also be noted that the SM used in this study had slightly lower total solids content than what has previously been reported for liquid milk from New Zealand herds (27). The lower total solids concentration (~80% of liquid sheep milk) was due to the reconstitution protocol used, which was according to the manufacturers' instructions. While the use of processed milk may have also impacted compositional features of the milks, including peptide structures, physiochemical properties, and digestion kinetics (20), there exist few comparisons of fresh pasteurized and powdered milk to inform how this processing technique (39) (particularly in the context of sheep milk) may have impacted the current findings. Thus, this study provides insight into the post-meal AA response to an equal serve of powdered SM relative to CM, but may not be predictive of differences in the digestion and postprandial AA appearance of sheep milk proteins if provided in quantities matched to cow milk, other regional variations in proportions, or differing processing techniques.

Although the current findings are a useful description of typical AA responses to differing ruminant species milks, it is important to note that the postprandial dynamics were described only in females, and further in dairy avoiders who generally restrict dairy consumption. These subjects, although self-described as avoiders, were largely lactose intolerant, yet many did consume some milk, albeit infrequently and in small amounts. Partial rather than complete avoidance has similarly been reported as more likely in Australian symptomatic dairy avoiders (1). This restriction of dairy, paired with intolerance, may have impacts on gastrointestinal transit and overall tolerance of milk, as habitual dairy consumption in lactose intolerance has been known to reduce malabsorption and intolerance (76, 77), including symptoms of flatulence (77). However, AA appearance in circulation has been shown to be unimpaired in subjects with lactose intolerance (78), although other forms of dairy intolerance may impact AA (78) and other nutrient appearance (79). While variation in dairy intake in the habitual diet may be expected to impact fasting amino acid profiles, we have previously demonstrated that changes to habitual dairy intake, such as restriction or increased intake over 1 month does not alter BCAA profiles, or indeed any other AA, in circulation (80). Yet, subjects who restricted dairy in the study by Prodhan et al. (80) still consumed 1.2 servings of dairy per day; as the current study did not record habitual dairy intake by means of a food frequency questionnaire or dietary records, it is unknown whether these participants had habitual intakes even below those previously reported. Indeed, others have reported that habitual protein intake, such as a high protein diet over 1 week, influences the postprandial AA response including N retention, albeit this effect has been shown to be more pronounced with soy intake rather than dairy (81). Hence, while the population studied here may have had alterations in habitual dairy intake or symptoms experienced with dairy relative to habitual consumers, it is less likely that this influenced postprandial AA appearance.

In summary, ingestion of SM and CM results in different responses when compared “portion-for-portion.” SM results in a greater circulating increase in the BCAAs leucine, isoleucine and valine, as well as the EAAs lysine and methionine and the non-essential AA proline compared to CM. These differences are not just a result of the compositional differences between SM and CM, but also reflect the inherent differences in protein digestion and amino acid absorption. The greater uptake of BCAAs may make SM an attractive, nutrient-dense alternative for consumers seeking bovine milk alternatives, including those who habitually avoid milk or have higher muscle maintenance requirements such as young children, the elderly and athletes. In addition, the greater uptake of lysine and methionine with SM may be beneficial for consumers of a vegetarian diet where these amino acids can be limiting.

## Data Availability Statement

The datasets presented in this article are not readily available because data described in the article, code book, and analytic code will not be made available because approval has not been granted by subjects. Requests to access the datasets should be directed to David Cameron-Smith, d.cameron-smith@auckland.ac.nz.

## Ethics Statement

The studies involving human participants were reviewed and approved by New Zealand Health and Disability Ethics Committees (Reference no. 18/NTB/92). The patients/participants provided their written informed consent to participate in this study.

## Author Contributions

AM designed and conducted research, analyzed data and performed statistical analysis, and wrote the paper. LS designed research, performed statistical analysis, and wrote the paper. AS conducted research, analyzed data, and wrote the paper. PS conducted research. LD designed research and wrote the paper. DC-S designed research and had primary responsibility for final content. All authors approved the final version of the manuscript for submission. All authors contributed to the article and approved the submitted version.

## Conflict of Interest

AM, LS, and LD are current employees of AgResearch Limited. The remaining authors declare that the research was conducted in the absence of any commercial or financial relationships that could be construed as a potential conflict of interest.
