# Supplementary material for: Circulating Branched Chain Amino Acid Concentrations Are Higher in Dairy-Avoiding Females Following an Equal Volume of Sheep Milk Relative to Cow Milk: A Randomized Controlled Trial
Source: Front Nutr. 2020 Nov 5;7:553674. doi: 10.3389/fnut.2020.553674 (PMC7678490; doi:10.3389/fnut.2020.553674)
Supplement: Supplementary file 1 [file Data_Sheet_1.docx]

Supplementary Material

# Supplementary Figures

**A**

**B**

**Supplemental Figure 1.** Typical reversed-phase HPLC chromatograms of plasma free amino acids. A: Plasma from participant 180 min after cow milk ingestion. B: Plasma from participant 180 min after sheep milk ingestion. Abbreviations: Asp - aspartic acid;Glu - glutamic acid; OH-Pro - hydroxyproline; Ser - serine; Asn - asparagine; Gly - glycine; Gln - glutamine; Tau - taurine; His - histidine; Cit - citrulline; Thr - threonine; Ala - alanine; Arg - arginine; Pro - proline; 1-MH - 1-methylhistidine; 3-MH - 3-methylhistidine; AABA - α-aminobutyric acid; Tyr - tyrosine; Val - valine; Met - methionine; Cys-Cys - cystine; Ile - isoleucine; Leu - leucine; Norleu - nor-leucine; Phe - phenylalanine; Trp - tryptophan; Orn - ornithine; Lys - lysine

**Supplementary Figure 2.** Postprandial changes in plasma essential amino acids not differing between sheep and cow milk following ingestion. Values presented as means ± SEM for histidine (A), phenylalanine (B), and threonine (C). There was a main effect of time for all presented amino acids (p < 0.05 each, respectively).

**Supplementary Figure 3.** Postprandial changes in plasma non-essential amino acids not differing between sheep and cow milk following ingestion. Values presented as means ± SEM for arginine (A), asparagine (B), aspartic acid (C), cystine (D), glutamic acid (E), glutamine (F), glycine (G), serine (H), and tyrosine (I). There was a main effect of time for all presented amino acids (p < 0.05 each, respectively).

**Supplementary Figure 4.** Postprandial changes in plasma non-proteogenic amino acids not differing between sheep and cow milk following ingestion. Values presented as means ± SEM for arginine (A), 1-methylhistidine (B), 2-methylhistidine (C), ɑ-aminobutyric acid (D), citrulline (E), hydroxyproline (F), ornithine (G), and taurine (H). There was a main effect of time for all presented amino acids (p < 0.05 each, respectively) except taurine (p > 0.05).
